# The 4-(Phenylsulfanyl) butan-2-one Improves Impaired Fear Memory Retrieval and Reduces Excessive Inflammatory Response in Triple Transgenic Alzheimer's Disease Mice

**DOI:** 10.3389/fnagi.2021.615079

**Published:** 2021-02-03

**Authors:** Peeraporn Varinthra, Kiruthika Ganesan, Shun-Ping Huang, Supin Chompoopong, Chatchakorn Eurtivong, Pavithra Suresh, Zhi-Hong Wen, Ingrid Y. Liu

**Affiliations:** ^1^Institute of Medical Sciences, Tzu Chi University, Hualien, Taiwan; ^2^Department of Molecular Biology and Human Genetics, Tzu Chi University, Hualien, Taiwan; ^3^Department of Anatomy, Faculty of Medicine Siriraj Hospital, Mahidol University, Bangkok, Thailand; ^4^Program in Chemical Sciences, Chulabhorn Graduate Institute, Chulabhorn Royal Academy, Bangkok, Thailand; ^5^Center of Excellence on Environmental Health and Toxicology (EHT), Commission on Higher Education (CHE), Ministry of Education, Bangkok, Thailand; ^6^Department of Marine Biotechnology and Resources, National Sun Yat-sen University, Kaohsiung, Taiwan

**Keywords:** Alzheimer's disease, inflammation, 3xTg-AD, fear conditioning, 4-(phenylsulfanyl) butan-2-one, natural compound, hippocampus, CA3

## Abstract

Alzheimer's disease (AD) is a neurodegenerative disease characterized by an excessive inflammatory response and impaired memory retrieval, including spatial memory, recognition memory, and emotional memory. Acquisition and retrieval of fear memory help one avoid dangers and natural threats. Thus, it is crucial for survival. AD patients with impaired retrieval of fear memory are vulnerable to dangerous conditions. Excessive expression of inflammatory markers is known to impede synaptic transmission and reduce the efficiency of memory retrieval. In wild-type mice, reducing inflammation response can improve fear memory retrieval; however, this effect of this approach is not yet investigated in 3xTg-AD model mice. To date, no satisfactory drug or treatment can attenuate the symptoms of AD despite numerous efforts. In the past few years, the direction of therapeutic drug development for AD has been shifted to natural compounds with anti-inflammatory effect. In the present study, we demonstrate that the compound 4-(phenylsulfanyl) butan-2-one (4-PSB-2) is effective in enhancing fear memory retrieval of wild-type and 3xTg-AD mice by reducing the expression of TNF-α, COX-2, and iNOS. We also found that 4-PSB-2 helps increase dendritic spine density, postsynaptic density protein-95 (PSD-95) expression, and long-term potentiation (LTP) in the hippocampus of 3xTg-AD mice. Our study indicates that 4-PSB-2 may be developed as a promising therapeutic compound for treating fear memory impairment of AD patients.

**Graphical Abstract:**
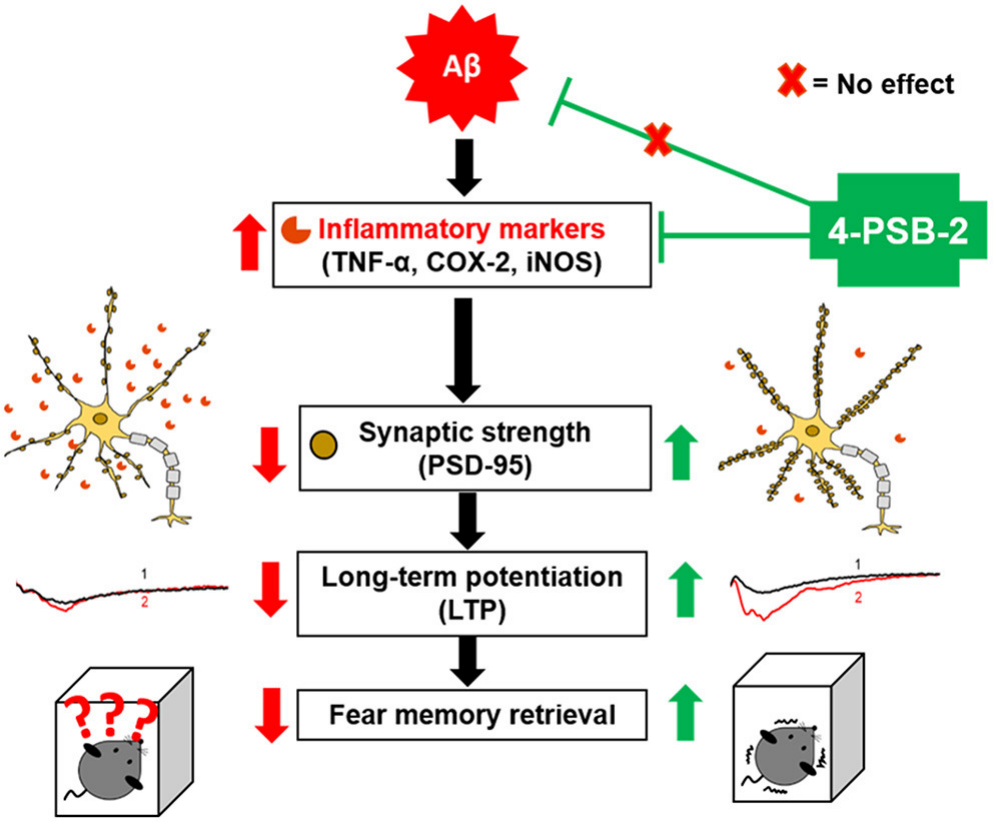
Schematic abstract. We are the first to report that 4-PSB-2 acts as memory enhancer and anti-inflammatory compound, which can reverse impairments in fear memory retrieval in 3xTg-AD mice without changing Aβ levels. 4-PSB-2 reduces the expression of several inflammatory markers including TNF-α, COX-2, and iNOS, increases PSD-95 expression in the hippocampus, and improves synaptic dysfunction in 3xTg-AD mice, which may account for its effect in improving fear memory retrieval.

## Introduction

Alzheimer's disease (AD) is a neurodegenerative disorder known to involve neuronal inflammation (Newcombe et al., 2018) and impaired memory retrieval, including episodic memory, recognition memory, spatial memory (Serrano-Pozo et al., 2011; Wahl et al., 2017), and fearful and traumatic memory (Hamann et al., 2002). Retrieval of fear memory elicits a fear response that helps avoid predators and natural threats. Thus, the fear response is important for survival, and the underlying neural circuits are highly conserved across species (Wotjak and Pape, 2013). Studies of AD patients have revealed that the fear response is defective at the onset stage of AD (Nasrouei et al., 2020). The AD model animals also exhibit impaired contextual fear memory (Billings et al., 2005; Kishimoto et al., 2017). An excessive inflammatory response is known to associate with impairments of working memory, remote memory stabilization, and spatial memory in AD (Murray et al., 2012; Wang et al., 2014; Mariani et al., 2017; Scuderi et al., 2018); however, its relationship with defective fear memory of AD is not clear yet.

The inflammatory response in the brain has been implicated in the initiation and progression of the pathogenesis of AD (Newcombe et al., 2018). The two AD pathological hallmarks, extracellular amyloid-beta (Aβ) deposition and intracellular neurofibrillary tangles, are known to increase inflammatory response and impede several types of memory functions, including episodic memory, recognition memory, semantic memory, spatial memory, and emotional memory (Serrano-Pozo et al., 2011; Klein-Koerkamp et al., 2012; Wahl et al., 2017). Early accumulation of Aβ and tau tangles can also induce astrogliosis and microglial activation, resulting in the elevated expression of proinflammatory mediators, including interleukin-1β (IL-1β), tumor necrosis factor-alpha (TNF-α), inducible nitric oxide synthase (iNOS), and interleukin-6 (IL-6) (Wang et al., 2015). Increases in these proinflammatory mediators can cause neurotoxicity, loss of synaptic transmission, reduced long-term potentiation (LTP) induction, and impaired cognitive functions in AD (Garwood et al., 2011; Fakhoury, 2018; Rajendran and Paolicelli, 2018). Previous studies have demonstrated that fear memory retrieval deficit is related to the decrease of LTP induction in AD mouse models such as Tg2576 mice (Comery et al., 2005), APP/PS1 mice (Gu et al., 2016), and 5XFAD mice (Kimura and Ohno, 2009). To date, no drug or treatment can cure this disease or effectively reduce the symptoms in humans. Most clinical trials of drugs targeting the clearance of Aβ have had limited success and failed to rescue memory functions (Morrison, 2016), while reducing inflammatory responses seem to delay the onset of AD symptoms (Akiyama et al., 2000; Businaro et al., 2018). The nonsteroidal anti-inflammatory drugs (NSAIDs) have been used to prevent or delay the onset of AD (Zhang et al., 2018). However, it was not effective and caused various adverse side effects including gastrointestinal bleeding, hypertension, and nephrotoxicity in elderly patients (Wongrakpanich et al., 2018). Therefore, finding a compound that can effectively prevent or treat fear memory impairment and reduce (excessive inflammatory response) in AD with less side effect is in an urgent need.

The compound 4-PSB-2 initially extracted from the soft coral *Cladiella australis*, has been shown to have anti-melanogenic effects via the suppression of tyrosinase activity in zebrafish embryos (Wu et al., 2015), and anti-inflammatory/neuroprotective effects with single-dose injection into a rat optic nerve crush model accompanied by reduced iNOS and cyclooxygenase-2 (COX-2) expression levels (Chien et al., 2016). Our recent study shows that Aβ_1−42_ oligomers-induced elevation of inflammatory markers in retinal pigment epithelial cells can be attenuated with a single-dose application of 4-PSB-2. Administration of this compound significantly suppressed expressions of TNF-α, COX-2, and iNOS (Varinthra et al., 2020). Besides, 4-PSB-2 is lipophilic; therefore, it is suitable as a drug candidate. According to all the 4-PSB-2 characteristics, we chose it to investigate whether reducing inflammation response in AD mice can improve impaired retrieval of fear memory. We injected the 4-PSB-2 into wild type and 3xTg-AD mice before fear memory testing. We found that this compound can effectively improve the deficit of contextual fear memory retrieval of the 3xTg-AD mice, reduce neuroinflammation response, and increase synaptic plasticity in the hippocampus.

## Methods

### Ethics Statement

All protocols used in this study were reviewed and approved by the Institutional Animal Care and Use Committee of Tzu Chi University (TCU, #108030), Taiwan and followed the guidelines of the Taiwan Ministry of Science and Technology on the ethical treatment of animals.

### Animals

Six-month-old male wild-type C57BL/6 mice with some mixed SV129 genetic markers, initially provided by the National Laboratory Animal Centre (Taiwan), were purchased and maintained undisturbed in the Laboratory Animal Centre of Tzu Chi University. Six-month-old 3xTg-AD mice were provided by Hei-Jen Huang from the Department of Nursing, Mackay Junior College of Medicine, Nursing and Management (Taiwan), and Hsiu Mei Hsieh-Li from the Department of Life Science, National Taiwan Normal University (Taiwan). The 3xTg-AD mice, initially purchased from the Jackson Laboratory (Stock #34830 JAX), harbor three mutations associated with AD and dementia in the genetic background of C57BL/6;129. The three mutations are two familial AD mutations (APP Swedish and PSEN1 M146V), and one mutation associated with frontotemporal dementia and parkinsonism-17 (tau P301L). Six-month-old male 3xTg-AD mice were used in this study. All mice were housed in individual plastic and metal cages in a temperature-controlled room with a 12-h light/dark cycle until the behavioral tasks were performed. Food and water were provided ad libitum.

### Preparation of 4-PSB-2 Solution and Treatment

**Figure 1:**
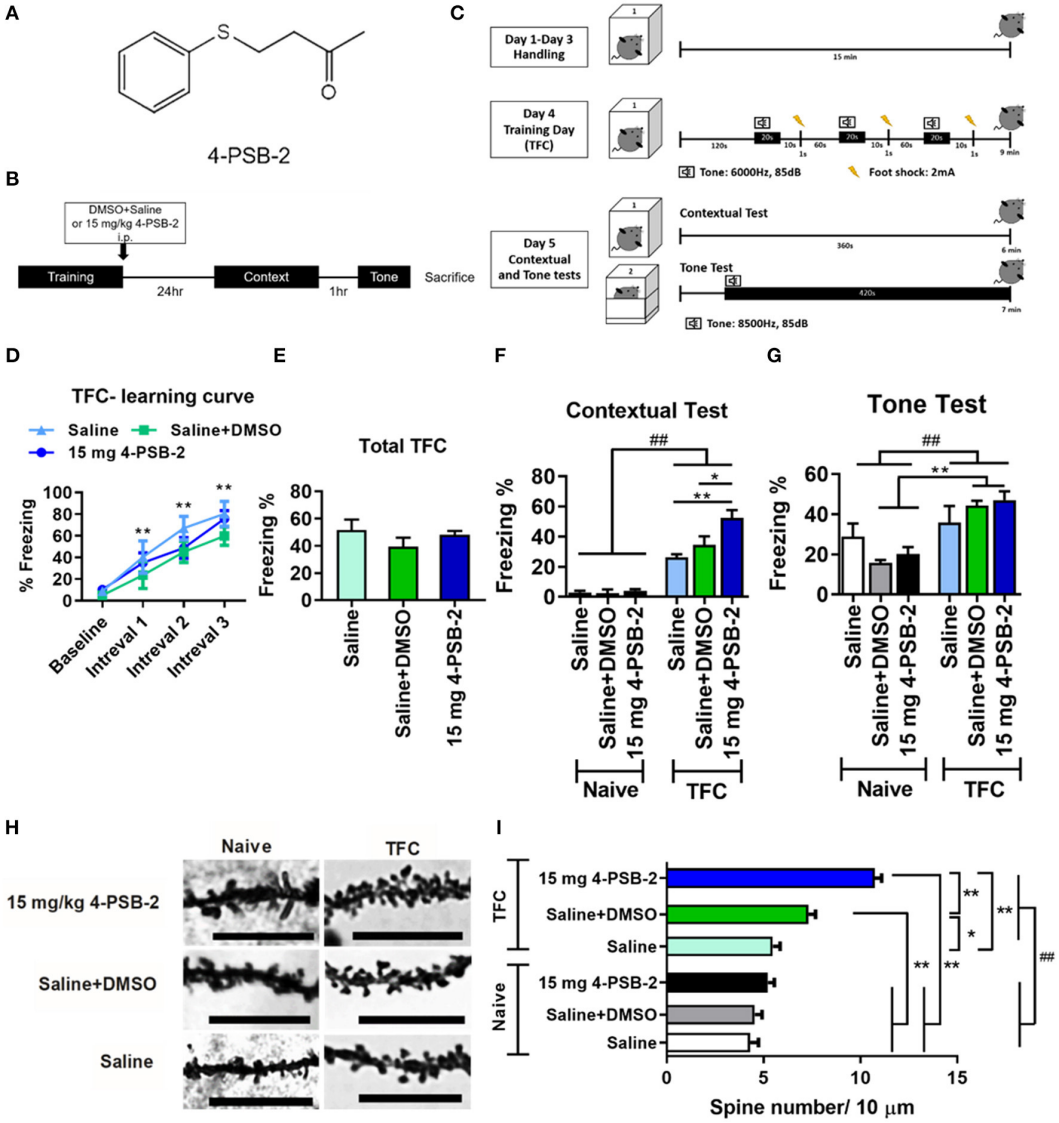
4-PSB-2 increased fear memory retrieval and dendritic spine density in the hippocampi of wild type (WT) mice. **(A)** The chemical structure of 4-PSB-2. **(B)** The timeline of 4-PSB-2 injection and TFC training. The WT mice were subjected to trace fear conditioning (TFC) and then immediately treated with 4-PSB-2. Contextual fear memory was tested 24 h later, and then a tone memory test was performed after a 1-h interval. **(C)** TFC protocol. The WT mice were handled in the conditioning chamber for 3 days before TFC. On day 4, the WT mice received 3 trials of TFC. Twenty-four hours later, their freezing responses to context were tested by placing them into the same conditioning chamber for 6 min, and then the tone memory test was performed in a different chamber for 6 min after a 1-h interval. **(D)** The WT mice acquired TFC when compared the freezing percentage at intervals 1–3 with baseline performance. **(E)** The results revealed that all groups were not significantly different in freezing percentage of total TFC. **(F)** There was a statistically significant interaction between the effects of TFC and treatments on freezing percentage change of contextual test in WT mice. The 4-PSB-2 injection at a concentration of 15 mg/kg significantly increased the retrieval of contextual fear memory and **(G)** slightly enhanced memory retrieval of a tone. **(H,I)** Among the trace fear-conditioned mice, the WT mice injected with 4-PSB-2 showed a significant increase in apical dendritic spine density. The number of WT mice in each group for TFC is shown. Naïve: saline (*n* = 4), saline + DMSO (*n* = 5), 15 mg 4-PSB-2 (*n* = 5); TFC: saline (*n* = 4), saline + DMSO (*n* = 5), 15 mg 4-PSB-2 (*n* = 5). *n* = 2/group for Golgi-COX staining. The results are shown in **(D–G)** and **(I)** are plotted as the means ± SEMs and were statistically analyzed by mixed-design repeated-measures ANOVA followed by Bonferroni test for **(D)**; two-way ANOVA followed by Tukey's test for **(F,G,I)**, ^##^indicates *p* ≤ 0.001 between the factors; one-way ANOVA followed by Tukey's test for **(E)**, *Indicates *p* ≤ 0.05, and **Indicates *p* ≤ 0.001 between the groups. Bar = 10 μm.

4-PSB-2 was provided by the Research Center of National Research Program for Biopharmaceuticals, Taiwan and its structure is shown in Figure 1A (Wen et al., 2014). 4-PSB-2 was dissolved in saline/DMSO at the ratio of 9:1. Three different concentrations (5, 10, and 15 mg/kg) of 4-PSB-2 were used in this study. 4-PSB-2 was intraperitoneally injected into mice immediately after trace fear conditioning (TFC).

### Trace Fear Conditioning

Wild-type (WT) mice were divided into two groups: (1) the WT group that did not undergo TFC and (2) the WT group that underwent TFC. Each group contained 3 subgroups (*n* = 5/subgroup), which included mice injected with (1) saline, (2) saline + dimethyl sulfoxide (DMSO), or (3) 15 mg/kg 4-PSB-2. 3xTg-AD mice (*n* = 8–11/group) were divided into three groups: (1) the untreated 3xTg-AD group, (2) sham 3xTg-AD group (saline + DMSO), and (3) the 3xTg-AD group treated with 15 mg/kg of 4-PSB-2. Fear conditioning was performed as described previously (Pai et al., 2018). On days 1-3, WT mice (*n* = 5/group) and 3xTg-AD mice were habituated to the conditioning chamber for 15 min per day. On day 4 (TFC day), the mice were placed in the chamber for 2 min and exposed to three trials of a tone (6,000 Hz, 85 dB; conditional stimulus) for 20 s followed by a 10-s interval and a 1-s foot shock (2 mA; unconditional stimulus). 4-PSB-2 was injected into mice immediately after TFC. Twenty-four hours later, the mice were placed in the same conditioning chamber for 6 min without the tone or foot shock for the contextual test. One hour later, the tone test was performed. The mice were placed in a new chamber for 1 min without a tone or foot shock and then exposed to a tone (6,000 Hz, 85 dB) for 6 min. Freezing behaviors were recorded and analyzed by FreezeScan software (CleverSys, Inc., VA, USA), and the freezing percentage was calculated as (total freezing time/total test time) × 100. Freezing behavior was characterized by the inhibition and absence of movement, heavy breathing, and minimal movement for normal respiration. Head scanning and sleeping were not considered freezing behaviors.

### Electrophysiological Recording

After TFC, the WT and 3xTg-AD mice were anesthetized and immediately sacrificed followed by taking the whole brain to incubate in ice-cold artificial cerebrospinal fluid (ACSF). Then, the hippocampus was horizontally sectioned for about 350 μm thickness by a vibrating microtome (Leica VT1000 S, Leica Biosystems Inc., Nussloch, Germany) in oxygenated (95% O_2_/5% CO_2_) ACSF. The hippocampal slices were incubated at 26°C for 2 h before recording. To induce LTP, the concentric bipolar tungsten electrodes and the recording glass pipettes with a micropipette puller were placed in the Schaffer collateral–commissural fibers at the stratum radiatum of the hippocampal CA1 region. During LTP inducing and recording, the slices were perfused continuously with ACSF at a speed of 20 rpm. The stimulation intensity was adjusted between 0 and 10 V for each slice. The field excitatory postsynaptic potentials (fEPSP) were elicited to approximately 50% of the maximal response. Before LTP induction, a steady baseline was recorded every 20 s for 20 min. LTP was evoked by high-frequency stimulation (HFS) for 3 trials of 100 Hz with a 20 s interval between each trial for 60 s. Then, fEPSPs were stimulated every 20 s for 60 min. The signals were amplified by an Axon Multiclamp 700B amplifier (Axon Instruments, Foster City, CA), acquired at 10 kHz by an Axon Digidata 1550B plus HumSilencer (Axon Instruments, Foster City, CA) and filtered at 1 kHz. The slope of fEPSPs is measured using Axon pCLAMP 11 electrophysiology data acquisition and analysis software.

### Western Blot Analysis

The mice were sacrificed by decapitation immediately after TFC to collect the hippocampi. The hippocampi were then homogenized in an ice-cold RIPA lysis buffer containing phosphatase and protease inhibitors (F. Hoffmann-La Roche AG, Basel, Switzerland). The samples were sonicated and centrifuged for 15 min at 13,500 × g at 4°C. The supernatants were collected, and the protein concentration was measured with the Bradford protein assay (Bio-Rad Laboratories, USA). Equal amounts of proteins from hippocampal tissues were separated by 10% sodium dodecyl sulfate–polyacrylamide gel electrophoresis (SDS-PAGE) and transferred to nitrocellulose membranes. The membranes were blocked with 1% bovine serum albumin (BSA) for 1 h at room temperature and incubated overnight at 4°C with the following primary antibodies: mouse anti-PSD-95 (1:2,000, MA1-045, Thermo Fisher Scientific) and mouse anti-β-actin (1:10,000, A5441, Sigma-Aldrich). After that, the membranes were washed three times with 1X phosphate-buffered saline (PBS) containing 0.1% Tween-20 and incubated with a horseradish peroxidase-conjugated (HRP) anti-mouse antibody (1:10,000, 7076, cell signaling technology) for 1 h at room temperature. The proteins of specific molecular weights were visualized using enhanced chemiluminescence reagents (Western Lightning® Plus-ECL, PerkinElmer, MA, USA) and detected by a UVP BioSpectrum 810 imaging system. Band intensity was quantified using ImageJ software (downloaded from National Institutes of Health, Bethesda, MD, USA, https://imagej.nih.gov/ij/download.html).

### Immunohistochemical Staining and Image Analysis

The hippocampi were taken at bregma −1.28 to −2.92 mm, then fixed with 4% paraformaldehyde (PFA) overnight at room temperature. After post-fixation, the brains were soaked in 30% sucrose at 4°C and cut coronally with cryostat every 20 μm. And then, the slides were fixed in methanol for 5 min at 4°C and dried at room temperature. Next, they were blocked with 1x PBS containing 2% BSA and 0.3% Triton X-100 for 2 h at room temperature and incubated with primary antibody: rabbit anti-TNF-alpha (1:300, ab6671, Abcam), rabbit anti-COX-2 (1:300, 12282, cell signaling technology), rabbit anti-iNOS (1:300, PA1-036, Thermo Fisher scientific), mouse anti-MAP2 (1:200, ab11267, Abcam), goat anti-GFAP (1:200, ab53554, abcam), goat anti-IBA1 (1:200, NB100-1028, Novus Biologicals), and rabbit anti-A11 (1:200, AHB0052, Thermo Fisher scientific), at 4°C, overnight. Sections washed in 1x PBS containing 0.3% Triton X-100 three times for 10 min and then incubated with the secondary antibody: Alexa 594 or Alexa 488 goat anti-rabbit IgG or goat anti-mouse (1:300, A-11037, A-11034, A-11056, A-11003, Thermo Fisher scientific, USA) or Donkey anti-goat IgG (1:300, ab150129, Abcam) for 1 h at room temperature in darkness. Sections were then again washed with 1x PBS containing 0.3% Triton X-100 three times for 10 min each, dried in room temperature, mounted with Fluoromount™ aqueous mounting medium, coverslipped and examined under fluorescence microscopy with 40X and 100X objective lens (Nikon ECLIPSE Ni-E). For calculating the positive area percentage of each antibody, 3–5 fields (200 × 200 μm) from 3 to 4 sections per mouse were quantified using ImageJ software (downloaded from National Institutes of Health, Bethesda, MD, USA, https://imagej.nih.gov/ij/download.html).

### Golgi-Cox Staining for Dendritic Spine Staining and Density Measurement

Mice were perfused transcardially with 0.9% saline followed by 4% PFA. After that, the whole brain was collected and fixed in 4% PFA overnight at room temperature. Next, the brains were transferred to Golgi-Cox solution and performed the staining process as previously described (Gibb and Kolb, 1998) for 5 days at 32°C. Then, the brains were transferred to 30% sucrose solution. The brains were sectioned at a thickness of 60 μm using a vibratome, and 5 sections/brain were collected for examination. Each group consisted of two animals, for each brain, 5 sections were chosen and 6 neurons from each section (3 from left, 3 from the right hippocampus—including DG and CA1) were selected for spine analysis. Neurons with properly stained dendrites were selected and the second to sixth proximal branch of apical dendrites were taken for spine counting. Spines were counted manually using ImageJ (downloaded from National Institutes of Health, Bethesda, MD, USA, website: https://imagej.nih.gov/ij/download.html). Spine density was represented as the number of spines per 10 μm of dendrite for 30–60 dendritic segments per group.

### Molecular Descriptor Calculation

The chemical structure of 4-PSB-2 was analyzed by using the Dragon 7.0 software suite (Kode Chemoinformatics, Pisa, Italy). Dragon 7.0 software is widely used to calculate molecular descriptors in scientific research (Kawczak et al., 2018), including molecular weight (MW); Moriguchi log P (*M*logP); hydrogen bond donors (HDs), and hydrogen bond acceptors (HAs), which are neighboring electronegative ions bearing a lone pair of electrons; the number of rotatable bonds (RBN); and total polar surface area (TPSA).

### Statistical Analysis

The mean ± standard error of the mean (mean ± SEM) of the data from this study was calculated and are plotted. The freezing percentage of total TFC, contextual and tone tests, locomotor activity, western blotting, immunohistochemical staining, and electrophysiological recording data in 3xTg-AD mice and WT –untreated were analyzed by one-way ANOVA followed by Tukey's *post hoc* test for multiple comparisons. The freezing percentage of contextual and tone tests, and synaptic spine density data in WT mice were analyzed the interaction of 2 factors including TFC and treatments on freezing percentage by two-way ANOVA followed by Tukey's *post hoc* test for multiple comparisons. The freezing percentage of the TFC learning curve of WT and 3xTg-AD mice were analyzed by mixed-design repeated-measures ANOVA followed by Bonferroni *post-hoc* test for multiple comparisons. Statistical significance of the differences among the groups was established at a *P*-value < 0.05. All graphs were plotted with GraphPad Prism 8.0 software.

## Results

### Chemical Structure Properties of 4-PSB-2

The compound 4-PSB-2 was initially extracted from a soft coral *Cladiella australis*, then modified and synthesized by the Development Center for Biotechnology (Taiwan), the National Research Program for Biopharmaceuticals support (CS-1-G-103-002). To investigate the chemical structure properties of 4-PSB-2 (Figure 1A) and its potential for use as a therapeutic agent, the Dragon 7.0 software suite was used. Descriptors were calculated based on Lipinski's rules and are listed in Table 1. Lipinski's rules are a set of indicators of a compound's oral bioavailability, including a MW < 500 g/mol, RBN < 10, *M*logP < 4.15, HD < 5, HA < 10, and TPSA < 140 Å2. From Table 1, it is clear that the 4-PSB-2 obeys Lipinski's rules and is likely to show acceptable oral bioavailability. In particular, 4-PSB-2 has low TPSA and high *M*logP, indicating that it is lipophilic; thus was chosen for use in this study.

**Table 1:** The calculated molecular descriptors of 4-PSB-2.

**Molecular Descriptors**	**4-PSB-2**
MW (g/mol)	180.29
RBN	4
*M*logP	2.723
HD	0
HA	1
TPSA (Å2)	42.37

*MW, Molecular weight; RBN, number of rotatable bonds; MlogP, Moriguchi log P; HDs, Hydrogen bond donors; HAs, Hydrogen bond acceptors; TPSA, Total polar surface area*.

### 4-PSB-2 Enhanced the Retrieval of Contextual Fear Memory and Increased Hippocampal Dendritic Spine Density in Wild-Type Mice

To investigate the effect of 4-PSB-2 on fear memory retrieval following TFC, mice were exposed to TFC training and then immediately treated with saline+DMSO or 4-PSB-2 (Figure 1B). Twenty-four hours later, a contextual fear memory was tested by returning the mice to the same conditioning chamber without any tone or foot shock. One hour after the contextual test, memory for the tone was tested by placing the mice in a different chamber and exposing them to tone cue for 6 min without delivering any electric footshock (Figure 1C). The percentage of freezing indicated fear memory retrieval of the context and tone. A higher freezing percentage indicated better memory retrieval. The WT mice acquired TFC when compared the freezing percentage at intervals 1–3 with baseline performance [*F*_(3, 33)_ = 38.767, *p* < 0.001; Figure 1D]. The results revealed that all groups were not significantly different in freezing percentage of total TFC [*F*_(2, 11)_ = 1.168, *p* = 0.347; Figure 1E]. There was a statistically significant interaction between the effects of TFC and treatments on freezing percentage change of contextual test in WT mice [*F*_(2, 22)_ = 6.136, *p* < 0.01]. Administration of 15 mg/kg 4-PSB-2 significantly enhanced the fear memory retrieval to context in WT mice [*p* < 0.001; Figure 1F]. There was a statistically significant interaction between the effect of TFC [*F*_(1, 22)_ = 31.045, *p* < 0.001] but not treatments [*F*_(2, 22)_ = 0.334, *p* = 0.72], on freezing percentage change of tone test in WT mice. Besides, the compound slightly enhanced fear memory retrieval to tone, although the effect was not statistically significant. Whereas, Naïve treated with saline + DMSO, Naïve treated with 15 mg/kg 4-PSB-2, TFC treated with saline + DMSO, and TFC treated with 15 mg/kg 4-PSB-2 groups showed a significant difference in fear memory retrieval to tone (*p* < 0.001; Figure 1G). 4-PSB-2 administration did not cause any impairment of locomotor activity, sociability, or anxiety in WT mice (Supplementary Material and Supplementary Figure 1).

To further verify the effect of 4-PSB-2 on memory retrieval in WT mice at the cellular level, dendritic spine density in the hippocampal region was evaluated using Golgi-Cox staining. The results indicated that at a concentration of 15 mg/kg, 4-PSB-2 significantly increased the apical dendritic spine density in the hippocampi of WT mice (Figure 1H). There was a statistically significant interaction between the effects of TFC and treatments on dendritic spine density in WT mice [*F*_(2, 283)_ = 18.65, *p* < 0.001]. Dendritic spine density of naïve mice was not changed after saline + DMSO and 4-PSB-2 treatments. After TFC, WT mice treated with saline + DMSO showed significantly increased dendritic spine density than naïve and the TFC + saline groups. Treatment with 4-PSB-2 at 15 mg/kg to WT mice after TFC significantly increased the dendritic spine density compared with naïve groups and TFC groups (*p* < 0.001; Figure 1I).

### 4-PSB-2 Injection Into 3xTg-AD Mice Rescued Impaired Fear Memory Retrieval to Context and Improved the Synaptic Dysfunction in 3xTg-AD Mice

**Figure 2:**
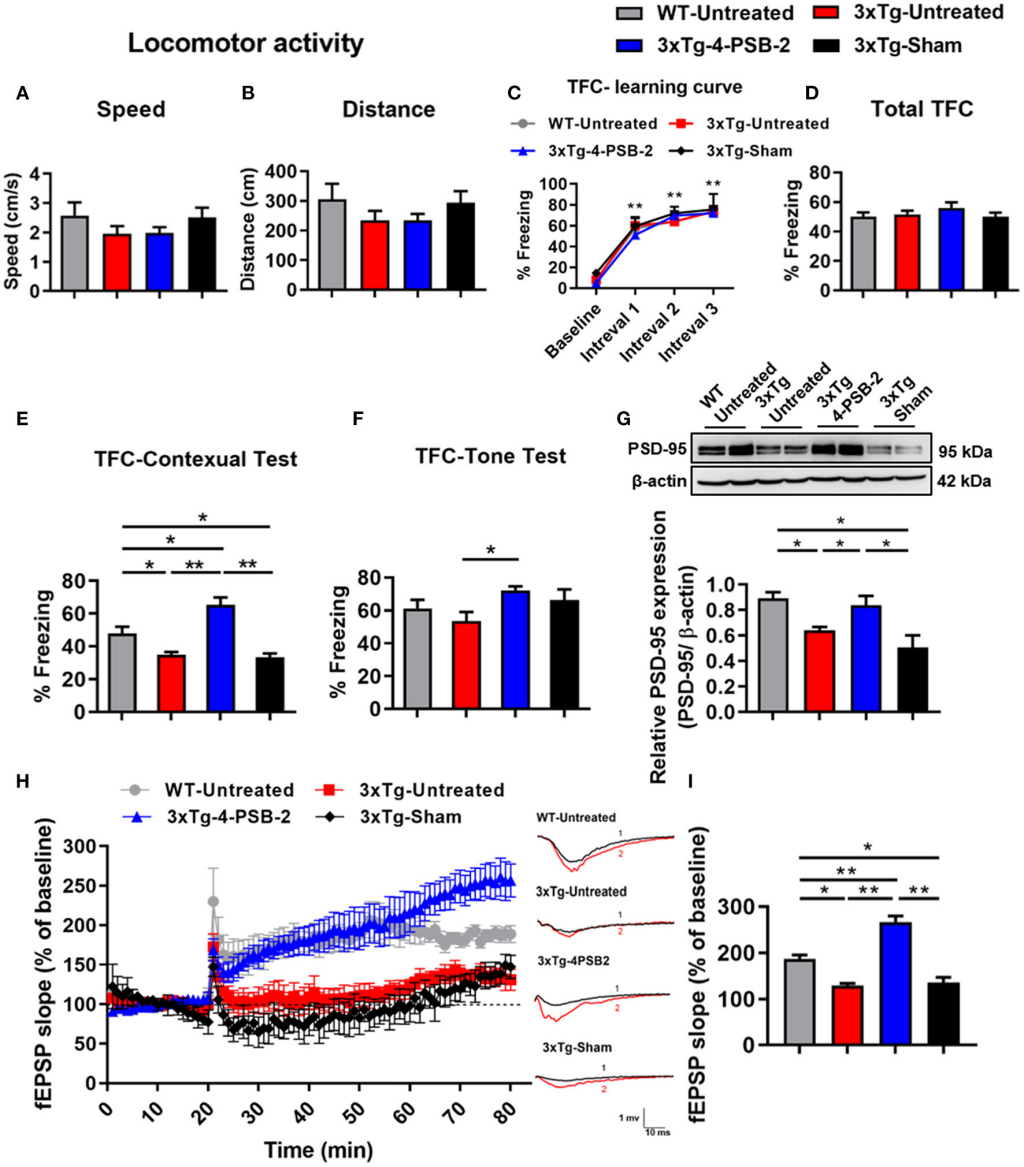
4-PSB-2 reversed contextual memory retrieval deficit and increased PSD-95 expression and synaptic plasticity in 3xTg-AD mice. **(A,B)** The locomotor activity of WT and 3xTg-AD mice was not significantly different before and after 4-PSB-2 injection. **(C)** The WT and 3xTg-AD mice acquired TFC when compared the freezing percentage at intervals 1–3 with baseline performance. **(D)** The 3xTg-AD mice were subjected to TFC training, and all groups appeared to learn normally. **(E)** The sham 3xTg-AD group exhibited reduced contextual memory retrieval compared to that of the untreated WT group, and the contextual memory retrieval and **(F)** memory retrieval of a tone of the 4-PSB-2-injected 3xTg-AD mice was significantly increased compared to that of the untreated 3xTg-AD mice. **(G)** Western blot analysis showed that PSD-95 expression levels in the hippocampi of the untreated 3xTg-AD and 3xTg-AD sham groups were significantly decreased compared to those in the untreated WT and were significantly increased after the administration of 4-PSB-2. **(H)** LTP was measured in the CA1 region of hippocampal sections of 3xTg-AD mice or WT mice with/without 4-PSB-2 administration for 60 min. **(I)** Last 10 min of the first hour after LTP induction, 4-PSB-2 injection showed restoration of LTP deficit in 3xTg-AD mice and also higher than WT mice. The results are plotted as the means ± SEMs and were statistically analyzed by mixed-design repeated-measures ANOVA followed by Bonferroni test for **(C)**; one-way ANOVA followed by Tukey's test for **(A,B)** and **(D–I)**. *Indicates *p* ≤ 0.05, and **Indicates *p* ≤ 0.001 between the groups; *n* = 8–11/group for TFC, *n* = 4–5/group for western blot analysis, and *n* = 3–5/group for LTP.

Since 4-PSB-2 at 15 mg/kg significantly enhanced the retrieval of contextual memory in WT mice (Figure 1F), we further tested whether it rescues impaired fear contextual memory in 3xTg-AD mice at this dose. 3xTg-AD mice exhibited Aβ deposition within neuronal and glial cells in the neocortex by 4 months and in the CA1 subregion of the hippocampus by 6 months (Oddo et al., 2003). The animals demonstrated impaired contextual fear and spatial memory at approximately 6 months of age (Billings et al., 2005). We injected 4-PSB-2 into 6-month-old 3xTg-AD mice immediately after TFC training and measured its effect on memory. After administration, 4-PSB-2 did not cause any impairment of locomotor activity in 3xTg-AD mice [*F*_(3, 34)_ = 1.048, *p* = 0.384; Figure 2A, *F*_(3, 34)_ = 1.09, *p* = 0.367; Figure 2B]. The WT-untreated and 3xTg-AD mice acquired TFC when compared the freezing percentage at intervals 1–3 with baseline performance [*F*_(3, 102)_ = 173.291, *p* < 0.001; Figure 2C]. The behavioral results demonstrated that the total freezing percentage of all groups after TFC was similar [*F*_(3, 34)_ = 0.828, *p* = 0.488; Figure 2D], but the retrieval of contextual memory was impaired in the 3xTg-AD mice compared to the untreated WT mice. Injection of 4-PSB-2 significantly enhanced the retrieval of contextual memory of the 3xTg-AD mice compared to that of the untreated 3xTg-AD and sham mice. Moreover, the 3xTg-AD mice treated with 4-PSB-2 showed a significantly higher level of freezing to context than the untreated WT group [*F*_(3, 34)_= 20.07, *p* < 0.001; Figure 2E]. Compared to the untreated WT mice, the 3xTg-AD mice did not show significantly impaired memory retrieval of the tone. However, 3xTg-AD mice treated with 4-PSB-2 exhibited significantly increased memory retrieval to tone [*F*_(3, 34)_ = 2.735, *p* = 0.059; Figure 2F].

As shown in Figure 1, 15 mg/kg 4-PSB-2 significantly increased the apical dendritic spine density in WT mice, we next asked what is the effect of 4-PSB-2 on PSD-95 expression in 3xTg-AD mice. Since PSD-95 is the major scaffolding protein at the excitatory postsynaptic density, a potent regulator of synaptic strength, and is known to be required for retrieval and stability of fear memory (Fitzgerald et al., 2015), we detected PSD-95 as an indirect synaptic marker in total protein samples extracted from the hippocampi of 3xTg-AD mice after TFC. Western blot analysis indicated that the PSD-95 expression level was significantly reduced in the untreated sham 3xTg-AD mice compared with the WT mice [*F*_(3, 15)_ = 7.601, *p* < 0.01; Figure 2G]. However, it was significantly enhanced after injection of 4-PSB-2 compared with that in the untreated and sham groups.

To further confirm the effect of 4-PSB-2 on synaptic plasticity in 3xTg-AD mice at the cellular level, LTP recording was performed after TFC. The results revealed that fEPSP after LTP induction in untreated 3xTg-AD and sham mice significantly decreased when compared with the untreated WT group. However, the injection of 4-PSB-2 significantly enhanced fEPSP after LTP induction of the 3xTg-AD mice compared to the untreated 3xTg-AD and sham mice. Furthermore, fEPSP after LTP induction in the 3xTg-AD mice treated with 4-PSB-2 showed a significantly higher than the untreated WT group [*F*_(3, 12)_ = 40.601, *p* < 0.001; Figures 2H,I].

### 4-PSB-2 Reduced the Expression Levels of Inflammatory Markers in the Hippocampal CA3 Region and Basolateral Amygdala

**Figure 3:**
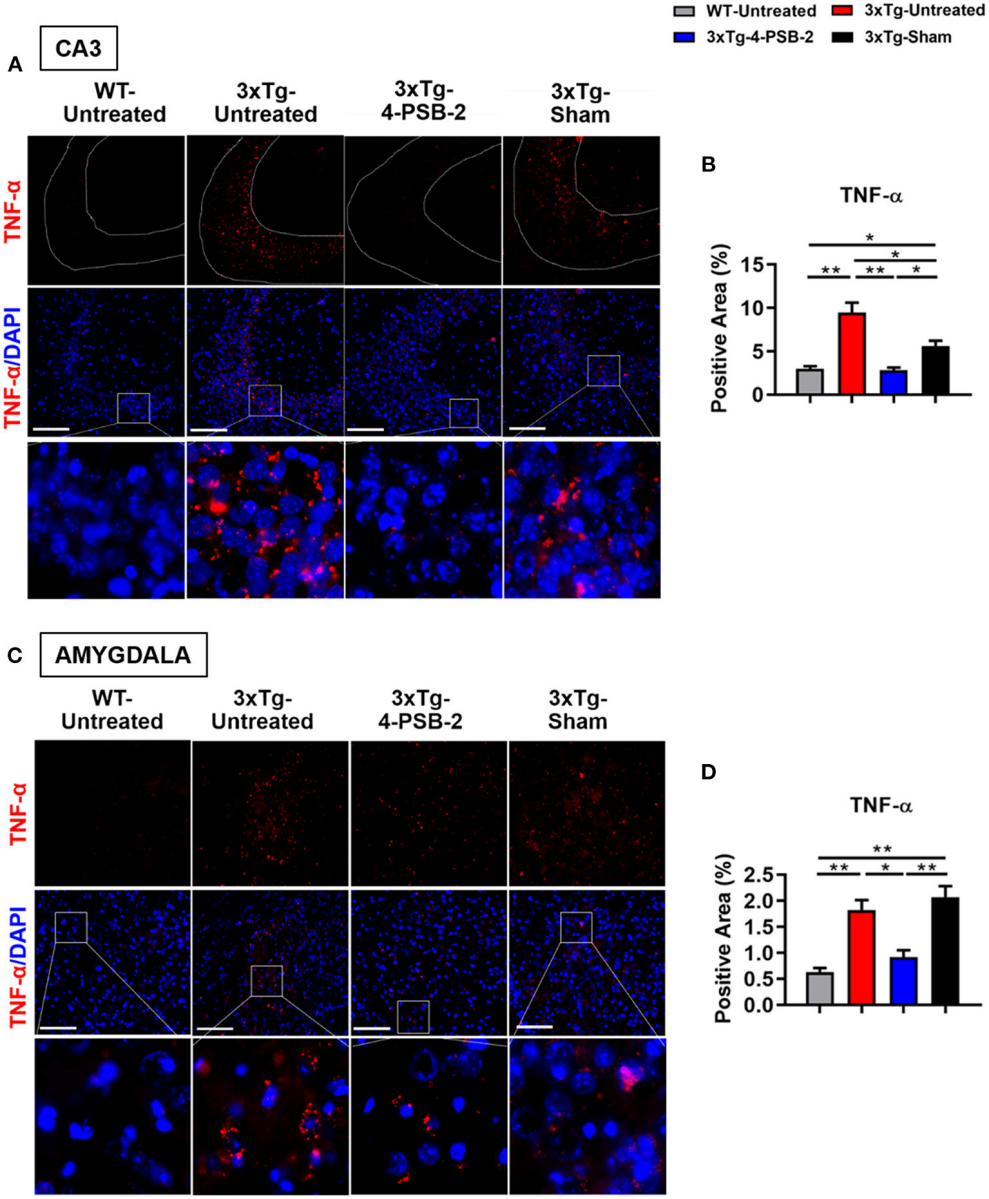
4-PSB-2 suppressed TNF- α expression in the hippocampal CA3 region and BLA of 3xTg-AD mice after TFC. **(A)** Immunofluorescence staining and **(B)** the quantitative result of TNF-α in the hippocampal CA3 region were significantly increased in the untreated 3xTg-AD and sham groups and were significantly decreased after 4-PSB-2 administration. **(C,D)** The expression of TNF-α in the BLA was significantly increased in the untreated 3xTg-AD and sham groups and was significantly decreased after the administration of 4-PSB-2. The results are plotted as the means ± SEMs. *Indicates *p* ≤ 0.05, and **Indicates *p* ≤ 0.001 between the groups. TNF-α (red) and DAPI (blue) (nuclei). Bar = 100 μm, and *n* = 3–5/group.

**Figure 4:**
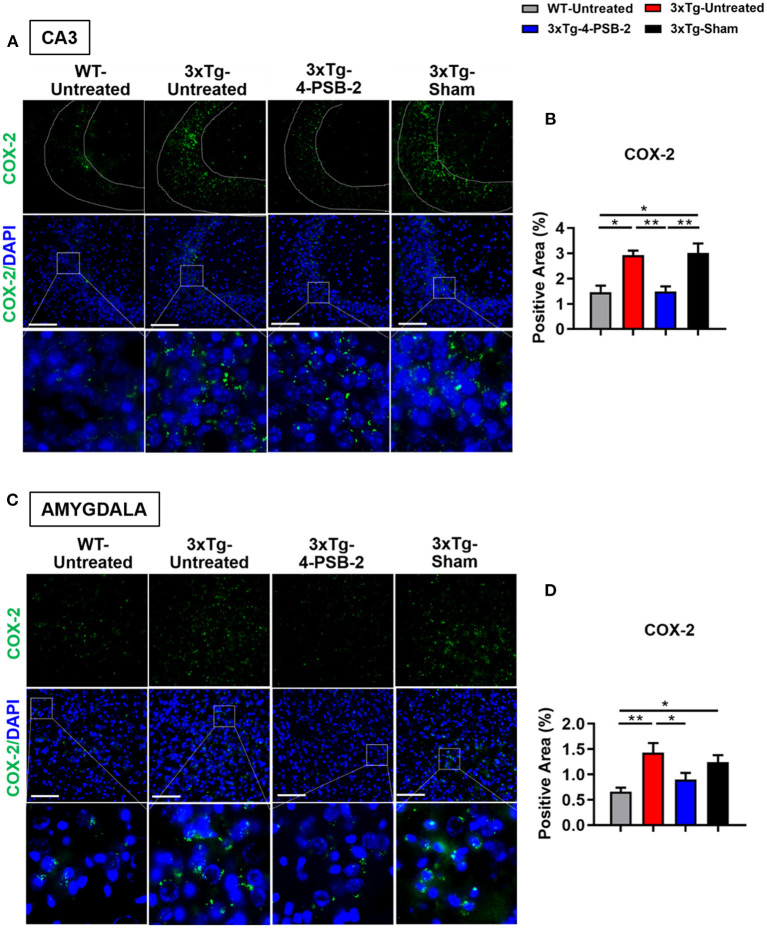
4-PSB-2 suppressed COX-2 expression in the hippocampal CA3 region and BLA of 3xTg-AD mice after TFC. **(A,B)** The expression of COX-2 in the hippocampal CA3 region was significantly increased in the untreated 3xTg-AD and sham groups and was significantly decreased after 4-PSB-2 administration. **(C,D)** The expression of COX-2 in the BLA of the untreated 3xTg-AD and sham groups were upregulated after TFC. After 4-PSB-2 administration, COX-2 expression was suppressed (compared with that in the untreated 3xTg-AD group). The results are plotted as the means ± SEMs. * indicates *p* ≤ 0.05, and **Indicates *p* ≤ 0.001 between the groups. COX-2 (green) and DAPI (blue) (nuclei). Bar = 100 μm, and *n* = 3–5/group.

**Figure 5:**
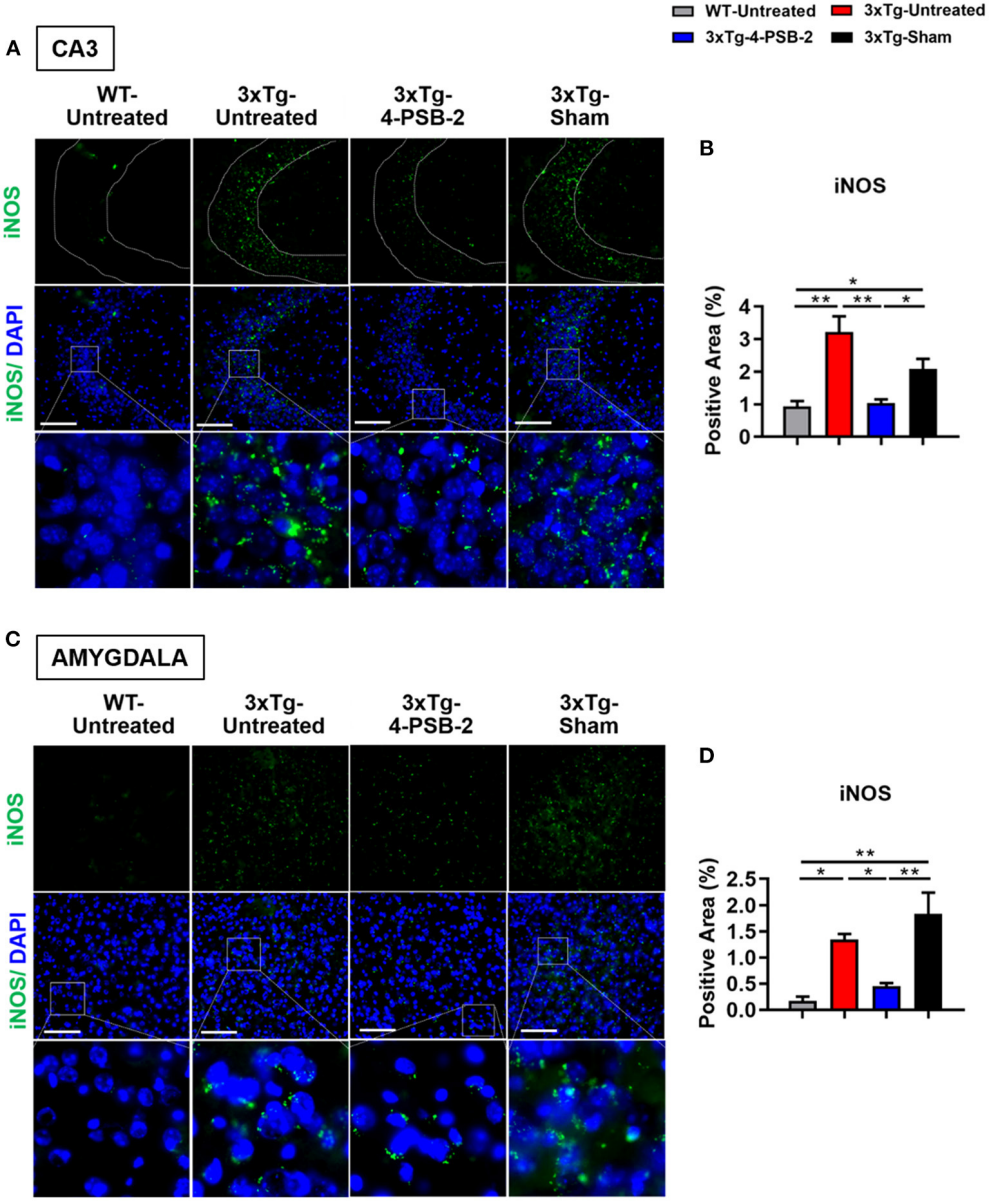
4-PSB-2 suppressed iNOS expression in the hippocampal CA3 region and BLA of 3xTg-AD mice after TFC. **(A,B)** The expression of iNOS in the hippocampal CA3 region was significantly increased in the untreated 3xTg-AD and sham groups and was significantly decreased after 4-PSB-2 administration. **(C,D)** The expression of iNOS in the BLA of the untreated 3xTg-AD and sham groups were upregulated after TFC. After 4-PSB-2 administration, iNOS expression was also suppressed (compared with that in the sham group). The results are plotted as the means ± SEMs. * indicates *p* ≤ 0.05, and **Indicates *p* ≤ 0.001 between the groups. iNOS (green) and DAPI (blue) (nuclei). Bar = 100 μm, and *n* = 3–5/group.

**Figure 6:**
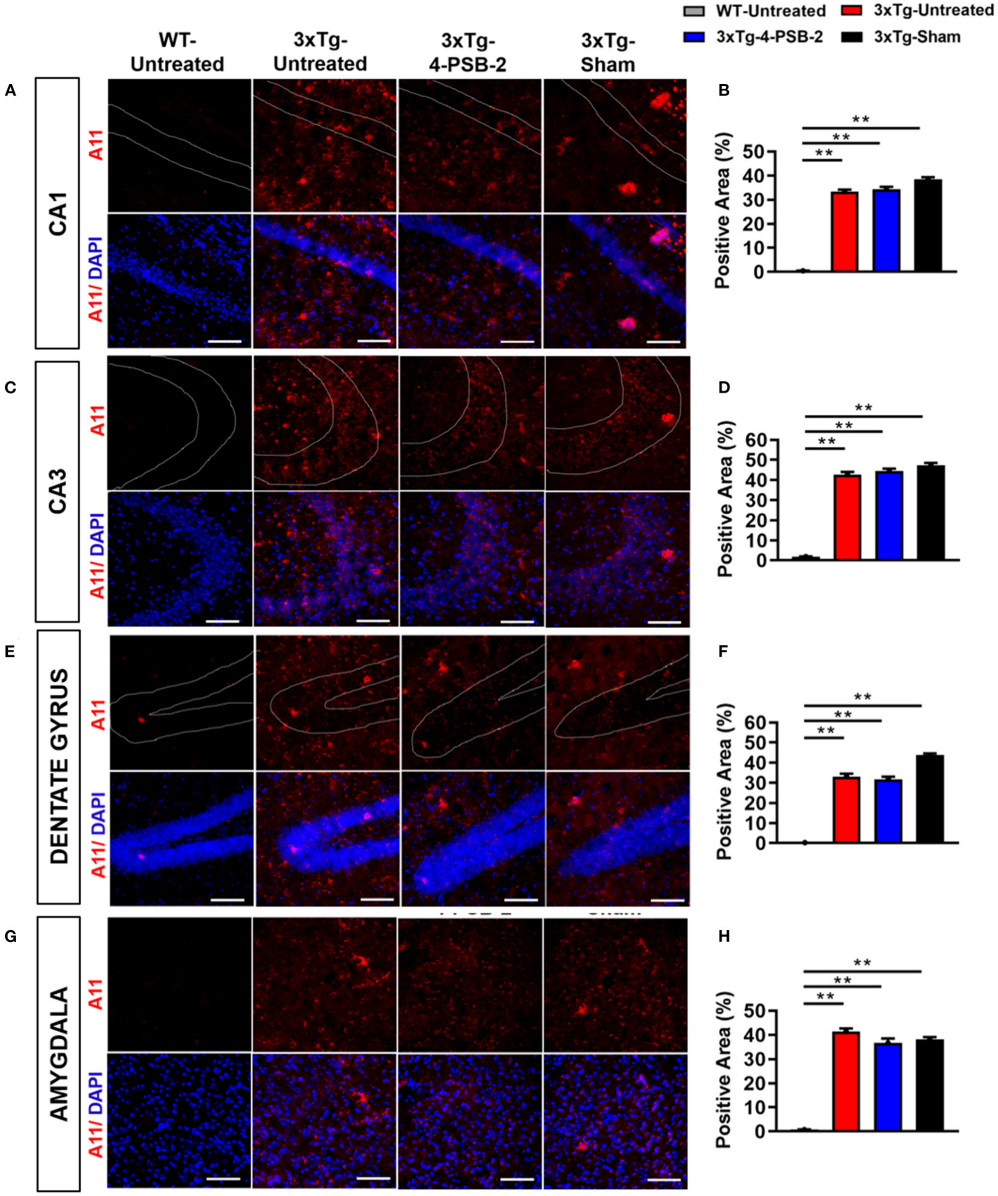
Expression of A11 in the hippocampus and BLA of 3xTg-AD mice after 4-PSB-2 treatment. Immunofluorescence staining of hippocampal **(A,B)** CA1, **(C,D)** CA3, **(E,F)** DG, and **(G,H)** BLA showed that despite the low expression level of A11 in the early AD mice brains, it was increased in all 3xTg-AD mice groups compared to the untreated WT group. After 4-PSB-2 treatment, the expression of A11 in 3xTg-AD mice was not different compared to the untreated 3xTg-AD and sham 3xTg-AD groups. The results are plotted as the means ± SEMs. **Indicates *p* ≤ 0.001 between the groups. A11 (red) = Aβ oligomer, and DAPI (blue) = nuclei, Bar = 100 μm.

Since 4-PSB-2 appeared to be effective in rescuing impaired memory retrieval in 3xTg-AD mice, we next asked whether memory retrieval is linked to expression changes in inflammatory markers in the involved brain region(s). We focused on the hippocampal region and basolateral amygdala (BLA) because these are the brain areas that play major roles in fear memory formation. The brains of WT and 3xTg-AD mice were collected after TFC and sectioned for immunohistochemical staining. The results revealed that in the hippocampal CA3 region, the expression levels of inflammatory molecules including TNF-α [*F*_(3, 55)_ = 40.375, *p* < 0.001; Figures 3A,B], COX-2 [*F*_(3, 41)_ = 9.779, *p* < 0.001; Figures 4A,B], and iNOS [*F*_(3, 58)_ = 11.68, *p* < 0.001; Figures 5A,B], were significantly increased in the untreated and sham 3xTg-AD group compared with the untreated WT mice. Administration of 15 mg/kg 4-PSB-2 significantly reduced the inflammatory cytokines expression levels. In the BLA, the expression levels of TNF-α [*F*_(3, 123)_= 17.713, *p* < 0.001; Figures 3C,D], COX-2 [*F*_(3, 47)_= 5.485, *p* < 0.01; Figures 4C,D], and iNOS [*F*_(3, 53)_= 8.982, *p* < 0.001; Figures 5C,D] were also increased in the untreated and sham 3xTg-AD groups and were reduced by 4-PSB-2 treatment. The expression levels of COX-2 but not TNF-α and iNOS in sham 3xTg-AD mice were significantly increased in the hippocampal CA1 region when compared with the untreated WT mice and were significantly decreased after 4-PSB-2 treatment. In the dentate gyrus (DG), the expression levels of COX-2 and iNOS but not TNF-α in untreated and sham 3xTg-AD mice were significantly increased, but were not decreased after 4-PSB-2 treatment (Supplementary Figures 2–4). We also detected the expression of Aβ oligomer (labeled with the A11 antibody) in the hippocampi and the BLA of 3xTg-AD mice. 3xTg-AD mice showed higher Aβ expression than WT mice in the hippocampal CA1 [*F*_(3, 87)_ = 20.968, *p* < 0.001; Figures 6A,B], CA3 [*F*_(3, 71)_= 14.669, *p* < 0.001; Figures 6C,D], dentate gyrus [*F*_(3, 73)_= 22.203, *p* < 0.001; Figures 6E,F], and BLA [*F*_(3, 62)_= 7.479, *p* < 0.001; Figures 6G,H], but 4-PSB-2 treatment did not change Aβ expression levels.

### Inflammatory Markers Were Expressed in the Cytoplasm of Neurons, Microglia, and Astrocytes in the Hippocampi of 3xTg-AD Mice After TFC

**Figure 7:**
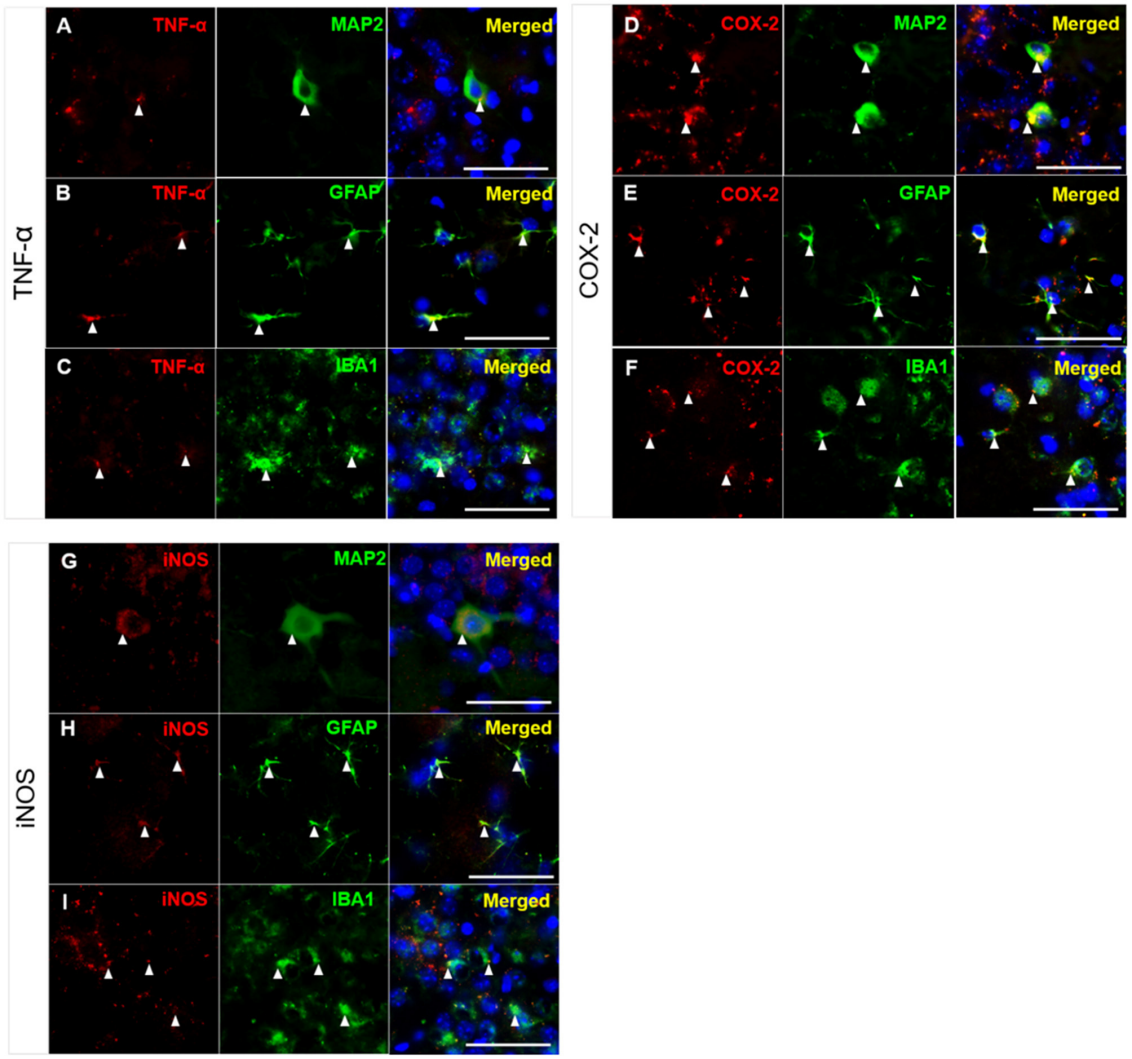
Colocalization of inflammatory molecules with neuronal and glial markers in CA3 region of 3xTg-AD mice. Double immunofluorescence staining for **(A–C)** TNF, **(D–F)** COX-2, and **(G–I)** iNOS with MAP2, GFAP, and IBA1 in the hippocampal CA3 region in 3xTg-AD mice. The results demonstrated that TNF-α, COX-2, and iNOS were detected in the cytoplasm of neurons (labeled with MAP2), astrocytes (labeled with GFAP), and microglial cells (labeled with IBA1), as indicated by the white arrowheads. TNF-α, COX-2 and iNOS (red); MAP2, GFAP, and IBA1 (green); and DAPI (blue) (nuclei). Bar = 50 μm.

As shown in Figures 4, 5, high levels of TNF-α, COX-2, and iNOS were detected in the hippocampal CA3 region in the untreated 3xTg-AD group after TFC. We next aimed to identify the cell types in which these inflammatory markers were expressed. The brains of 3xTg-AD mice were collected after TFC for double staining with inflammatory markers (TNF-α, COX-2, and iNOS), a neuronal marker (MAP2) or glial markers (GFAP for astrocytes and IBA1 for microglia). TNF-α was colocalized with MAP2 (Figure 7A), GFAP (Figure 7B), and IBA1 (Figure 7C) in the cytoplasm. TNF-α downstream targets, COX-2 and iNOS (Song et al., 2012), were also found to be colocalized with MAP2 (Figures 7D,G), GFAP (Figures 7E,H), and IBA1 (Figures 7F,I) in the cytoplasm. The results indicate that these inflammatory markers were expressed in all three cell types.

## Discussion

In the present study, we report that 4-PSB-2 significantly rescues the impairment of fear memory retrieval in 3xTg-AD mice and increases dendritic spine density and LTP through the suppression of several inflammation markers in the BLA and hippocampus (Graphical Abstract), particularly in the CA3-region. Studies on AD patients have confirmed that atrophy (Basso et al., 2006) and functional disconnectivity (Ortner et al., 2016) of the hippocampus and BLA are associated with memory decline (Basso et al., 2006; Ortner et al., 2016). These two brain regions are known to be important for the retrieval and mediation of fear memory in non-AD mice (Anagnostaras et al., 2001; Gale et al., 2004; Lu et al., 2009). More internal synaptic connections existing in the hippocampal CA3-region than in other regions has been reported (Cherubini and Miles, 2015). Therefore, the loss of neurons and synaptic connectivity within the CA3-region may reduce the precision of memory retrieval (Chadwick et al., 2014) in AD.

We further found that the inflammatory markers in the CA3-region were expressed in neurons, microglia, and astrocytes of 3xTg-AD mice. These inflammatory molecules seem to mediate cross-talk among these three types of cells. Microglia are glial cells that are responsible for homeostasis and immune defenses in the brain and can become polarized toward the proinflammatory M1 and immunosuppressive M2 phenotypes by Aβ deposition (Fakhoury, 2018). In AD, an excess of Aβ deposition disrupts the balance between M1 and M2 microglia, resulting in the overexpression of proinflammatory molecules (IL-1β, TNF-α, iNOS, and IL-6), in turn causing neuronal damage (Tang and Le, 2016). The increase of proinflammatory molecules such as LPS, IL-1β, TNF-α, and IFN-γ also interfered with the microglia's capacity to remove Aβ by suppressing microglial endocytic (Sole-Domenech et al., 2016) and phagocytic activities (Koenigsknecht-Talboo and Landreth, 2005). The microglial endocytosis can be improved by applying 40 Hz gamma oscillations to the brains of AD mice (Iaccarino et al., 2016). The microglial phagocytic activity can be improved with anti-inflammatory treatment (Koenigsknecht-Talboo and Landreth, 2005). Astrocytes are also activated by Aβ and then secrete excessive inflammatory cytokines leading to neuronal injury (Fakhoury, 2018). Several studies have revealed that inflammatory cytokines such as the TNF-α (Yu et al., 2017), COX-2 (Chen et al., 2013), and iNOS (Lisboa et al., 2015) played important roles in regulating contextual fear memory. It is possible that in AD or aging mice, the inflammatory response is enhanced and lasts for long, which leads to over-activation of microglia and astrocytes in the CA3-region, resulting in neuronal function deficit, synaptic loss, synaptic transmission inefficiency (Mariani et al., 2017), and learning and memory impairment (Lana et al., 2016). Moreover, the brain areas other than the CA3-region and BLA are also affected by an enhanced inflammatory response in this transgenic AD mouse strain upon aging (Backman et al., 1999; Kinney et al., 2018). Therefore, more age-dependent memory tests need to be performed in AD mice to elucidate the relationship between changes of the inflammatory response in different brain regions.

The present study shows that 4-PSB-2 acts as a memory enhancer to increase contextual fear memory, elevate dendritic spine density, PSD-95 expression, and LTP in the hippocampus. The PSD-95 is a key protein that increases synaptic strength and the efficiency of the formation and retrieval of fear memory (Hering and Sheng, 2001; Fitzgerald et al., 2015; Huang et al., 2018). It is also known to regulate glutamatergic plasticity at the postsynaptic site (El-Husseini et al., 2000) and stabilizes α-amino-3-hydroxy-5-methyl-4-isoxazolepropionic acid receptors (AMPARs) to promote synaptic function and spine growth (Chen et al., 2011). The reduction of PSD-95 and synaptic spine density can interfere with hippocampal LTP in AD mouse models (Tu et al., 2014; Gu et al., 2016). LTP is a well-accepted cellular representation of the synaptic plasticity indicating the efficiency of learning and memory. In the present study, we recorded reduced LTP from the Schaffer-collateral pathway, which sends efferent fiber from the CA3 to the CA1 regions of the hippocampus (Kumar, 2011; Cherubini and Miles, 2015). This phenomenon is in line with previous findings from other AD mouse models such as Tg2576 mice (Comery et al., 2005), APP/PS1 mice (Gu et al., 2016), and 5XFAD mice (Kimura and Ohno, 2009). Interestingly, the impaired LTP was reversed by 4-PSB-2 administration that has anti-inflammatory effects, indicating a relationship between levels of inflammation response with synaptic transmission.

The 4-PSB-2 has a similar chemical structure as BAY 11-7082 reported in a previous study (Lee et al., 2012), which can also suppress the expression of inflammatory proteins, including IκB, NF-κB, and Akt. The structure of BAY 11-7082 varies slightly from that of 4-PSB-2, as it contains a reactive Michael acceptor and a cyanide group in place of a ketone group. The structural similarities of the compounds probably underlie their similar functions in suppressing the inflammatory response. The 4-PSB-2 is likely able to pass through the blood-brain barrier since it is lipophilic and small; its molecular weight (180) is <400 Daltons (Pardridge, 2012). Our results are consistent with previous studies (Moy et al., 2004; Nakatani et al., 2009; Pearson et al., 2010), in which the “social recognition” of C57BL/6 remained intact with alterations to the “social novelty” behavior. The C57BL/6 mice in our study showed an increasing trend in “social novelty” behavior though not significant. This behavior was improved after 4-PSB-2 treatment. The 4-PSB-2 was administered immediately after TFC because it is important for memory consolidation and inflammatory response induction (Barrientos et al., 2002; Johansen et al., 2011; White et al., 2020). Efficient memory consolidation in the hippocampus is known to help better memory retrieval (Bosshardt et al., 2005). Post-injection behavioral evaluation did not indicate any effect that 4-PBS-2 seems to cause on anxiety test and locomotor activity of WT and 3xTg-AD mice, which suggests that 4-PSB-2 may be a good drug lead. It is noted that a single dose of 4-PSB-2 used in this study did not significantly reduce Aβ oligomer expression in the hippocampus (Shin et al., 2014) and BLA of 3xTg-AD mice; therefore, further time course studies of pharmacokinetics, toxicity, metabolism, and the effects of multiple doses are necessary.

In conclusion, the excessive inflammatory response may be associated with impairment of fear memory retrieval occurred in early-stage AD. Our results demonstrate that targeting and reducing the inflammatory response in the brain with the 4-PSB-2 may be a promising approach to prevent and treat related memory symptoms of AD.

## Data Availability Statement

The original contributions presented in the study are included in the article/Supplementary Material, further inquiries can be directed to the corresponding author/s.

## Ethics Statement

The animal study was reviewed and approved by The Institutional Animal Care and Use Committee of Tzu Chi University.

## Author Contributions

IL and PV developed hypotheses, designed and performed experiments, analyzed data, prepared Figures 2–7, Graphical Abstract, and wrote manuscript. KG developed hypotheses, performed the experiment, analyzed data, and prepared Figure 1. S-PH and SC developed hypotheses, designed experiments, and helped technical support. CE performed the experiment, analyzed data, and prepared Figure 1A and Table 1. PS helps with experimental designs, performed the experiment, and prepared the manuscript. Z-HW provided the compound and helped interpret results. All authors read and approved the final version of this manuscript.

## Conflict of Interest

The authors declare that the research was conducted in the absence of any commercial or financial relationships that could be construed as a potential conflict of interest.
