# Supplementary material for: The 4-(Phenylsulfanyl) butan-2-one Improves Impaired Fear Memory Retrieval and Reduces Excessive Inflammatory Response in Triple Transgenic Alzheimer's Disease Mice
Source: Front Aging Neurosci. 2021 Feb 3;13:615079. doi: 10.3389/fnagi.2021.615079 (PMC7888344; doi:10.3389/fnagi.2021.615079)
Supplement: Supplementary file 1 [file Data_Sheet_1.docx]

Supplementary Material

# Supplementary Methods

**Open field test**

Mice were placed in an open chamber (50 cm x50 cm x50 cm) with no cue or stimulus for 10 minutes and were allowed to freely move around the chamber. A video camera and tracking device (Track Mot, Drinstrument, Taiwan) were used to measure its locomotor activity in the chamber. The distance and speed were calculated.

**Three chamber test**

Three chamber test is carried out to assess the social behavior of the mice. After the injection of 4-PSB-2, mice were taken for three trials during this test. In the beginning, mice were placed in the middle of the three chambers for each trial. ***Trial 1***: The mice were allowed to explore the three chambers for about 10 minutes. A video camera and tracking device (Track Mot, Drinstrument, Taiwan) were used to measure its exploring behavior activity. ***Trial 2***: A stranger mouse was placed in a small cage of right chamber and the mice were again placed in the chamber for 10 minutes. ***Trial 3***: Two stranger mice 1 and 2 were placed in a small cage of right chamber and left chamber, respectively, and the experimental mice were placed in the chamber for 10 minutes. The stranger mice 1 was not changed between the trials 2 and 3. The social behavior of mice was assessed by the time spent with the stranger mice. The social recognition/sociability is assessed through trial-2 where the mice are expected to spend an increased amount of time with the new stranger mouse (stranger 1) compared to an empty cage. Social novelty can be assessed in trial 3 where it is expected that the mice spend a significant amount of time with the new stranger mouse (stranger 2) compared to the familiar mouse (stranger 1).

**Elevated plus maze test (EPM)**

EPM was used to measure the anxiety levels of mice. It was performed by placing the mice in an elevated (60 cm from the ground) plus maze for 10 minutes. The plus maze has 2 closed arms and 2 open arms opposite to each other. A video camera and tracking device (Track Mot, Drinstrument, Taiwan) were used to measure its activity. Anxiety behavior of mice was assessed by the time spent in the closed and open arms.

# Supplementary Figures and Tables

## Supplementary Figure 1

##
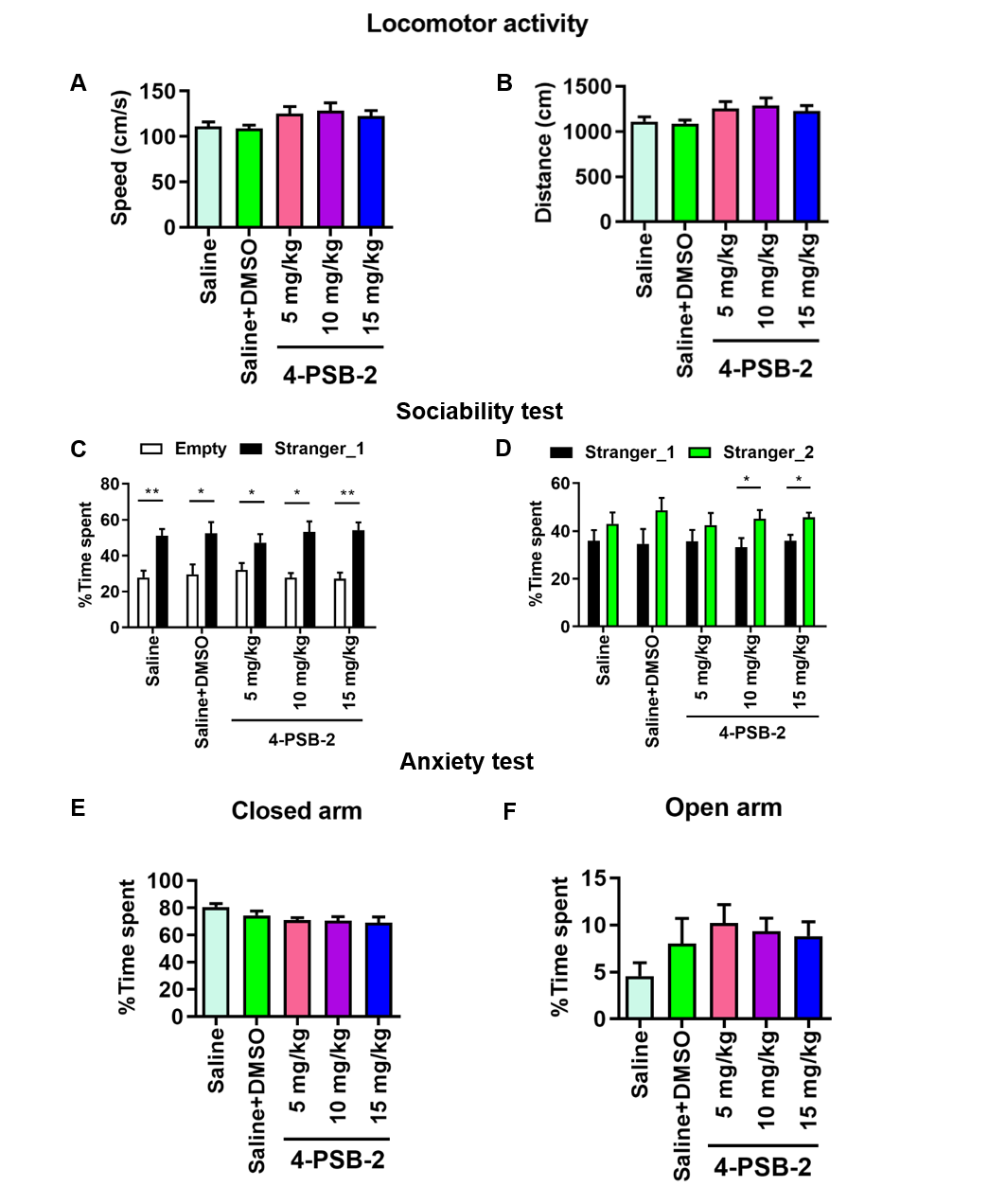


**Supplementary Figure 1.** 4-PSB-2 did not affect the behavioral activities of wild type mice. The graph shows the behavioral activities of wild type mice after 5, 10, and 15 mg/kg of 4-PSB-2 injection compared to saline and saline+DMSO groups. **(A)** The speed and **(B)** moving distance of wild type mice for 10 minutes after 4-PSB-2 injection. The sociability test showed that **(C)** all groups exhibited normal social recognition behavior by spending increased time with stranger 1 compared to that with the empty cage, and **(D)** wild type mice treated with saline and saline+DMSO did not exhibit “social novelty” behavior, which was improved by 5, 10, and 15 mg/kg of 4-PSB-2 treatment. The anxiety test demonstrated the time spent of wild type mice in **(E)** closed arm and **(F)** open arm after 4-PSB-2 administration. Statistics: one-way ANOVA; **A**, *F*_(4,25)_= 1.957, *p*= 0.132, **B**, *F*_(4,25)_= 1.966, *p*=0.131, **E**, *F*_(4,25)_= 2.185, *p*=0.1, **F**, *F*_(4,25)_= 1. 374, *p*=0.271. Statistics: unpaired T-test; **C**, saline, *t*_(10)_= -4.399, *p*<0.001, saline+DMSO, *t*_(10)_= -2.724, *p*<0.05, 5mg/kg, *t*_(10)_= -2.764, *p*<0.05, 10mg/kg, *t*_(10)_= -3.972, *p*<0.01, 15mg/kg, *t*_(10)_= -4.531, *p*<0.001, **D**, saline, *t*_(10)_= -1.099, *p*=0.297, saline+DMSO, t_(10)_= -1.737, *p*=0.113, 5mg/kg, *t*_(10)_= -0.963, *p*=0.358, 10mg/kg, *t*_(10)_= -2.268, *p*<0.05, 15mg/kg, *t*_(10)_= -3.119, *p*<0.05. The results were plotted as the means±SEMs and statistically tested with one-way ANOVA followed by Tukey’s Test for locomotor activity and anxiety test or unpaired T-test for sociability test, * indicates *p* ≤0.05, and ** indicates *p*≤ 0.01 between the groups (n=5-6/group).

## Supplementary Figure 2


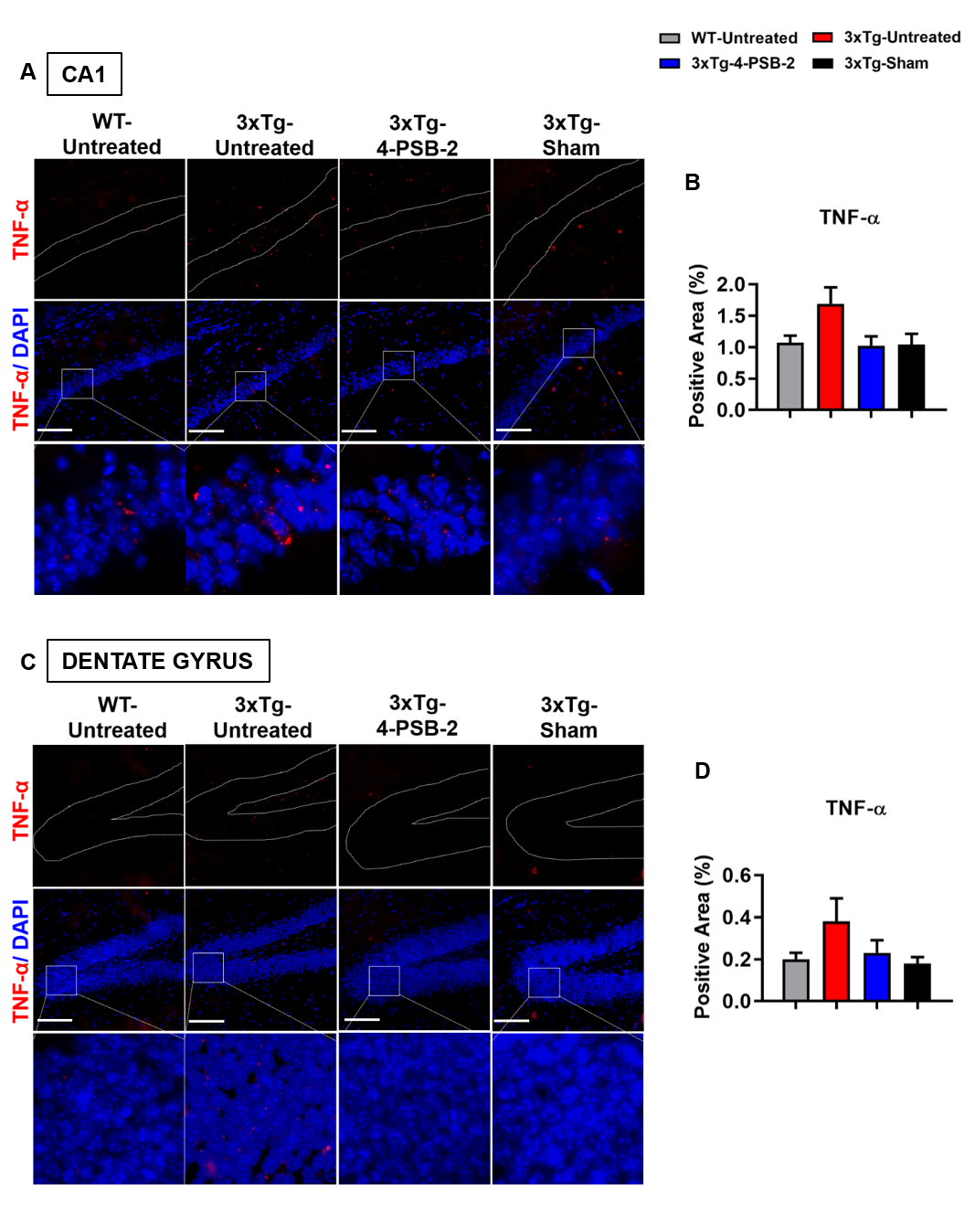


**Supplementary Figure 2.** Expression of TNF- α in the hippocampal CA1-region and dentate gyrus of 3xTg-AD mice after TFC. **(A, C)** Immunofluorescence staining and **(B, D)** the quantitative results of the hippocampal CA1-region and dentate gyrus in 3xTg-AD mice showed that the expression levels of TNF-α were low and not significantly different among groups. Statistics: one-way ANOVA; **B**, *F*_(3,136)_= 3.33, *p*=0.021; **D**, *F*_(3,60)_= 3.007, *p*=0.037. The results are plotted as the means±SEMs, * indicates *p* ≤0.05, and ** indicates *p*≤ 0.001 between the groups. TNF-α (red) and DAPI (blue) = Nucleus, Bar= 100 µm.

## Supplementary Figure 3

##
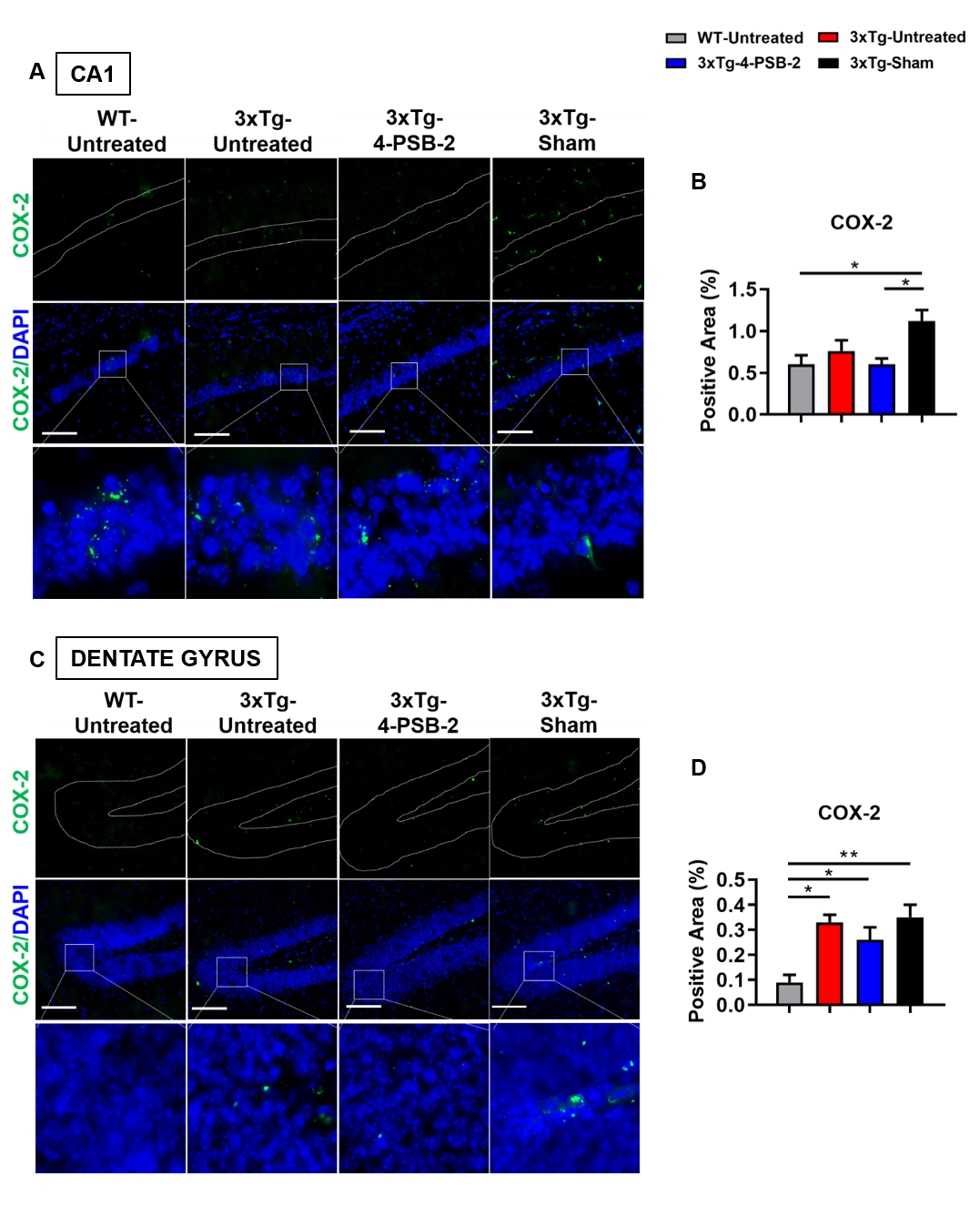


**Supplementary Figure 3.** Expression of COX-2 in the hippocampal CA1-region and dentate gyrus of 3xTg-AD mice after TFC. **(A-B)** Immunofluorescence staining and the quantitative results of the hippocampal CA1-region in 3xTg-AD mice showed that the expression levels of COX-2 in the 3xTg-AD-sham group was significantly increased after TFC compared with WT-untreated group, and decreased when administration with 4-PSB-2. **(C-D)** The COX-2 expression levels in dentate gyrus were significantly increased in the 3xTg-AD-untreated, sham, and 4-PSB-2 groups. Statistics: one-way ANOVA; **B**, *F*_(3,76)_= 3.190, *p*=0.028; **D**, *F*_(3,38)_= 6.611, *p*<0.001. The results are plotted as the means±SEMs, * indicates *p* ≤0.05, and ** indicates *p*≤ 0.001 between the groups. COX-2 (green) and DAPI (blue) = nuclei, Bar= 100 µm.

## Supplementary Figure 4


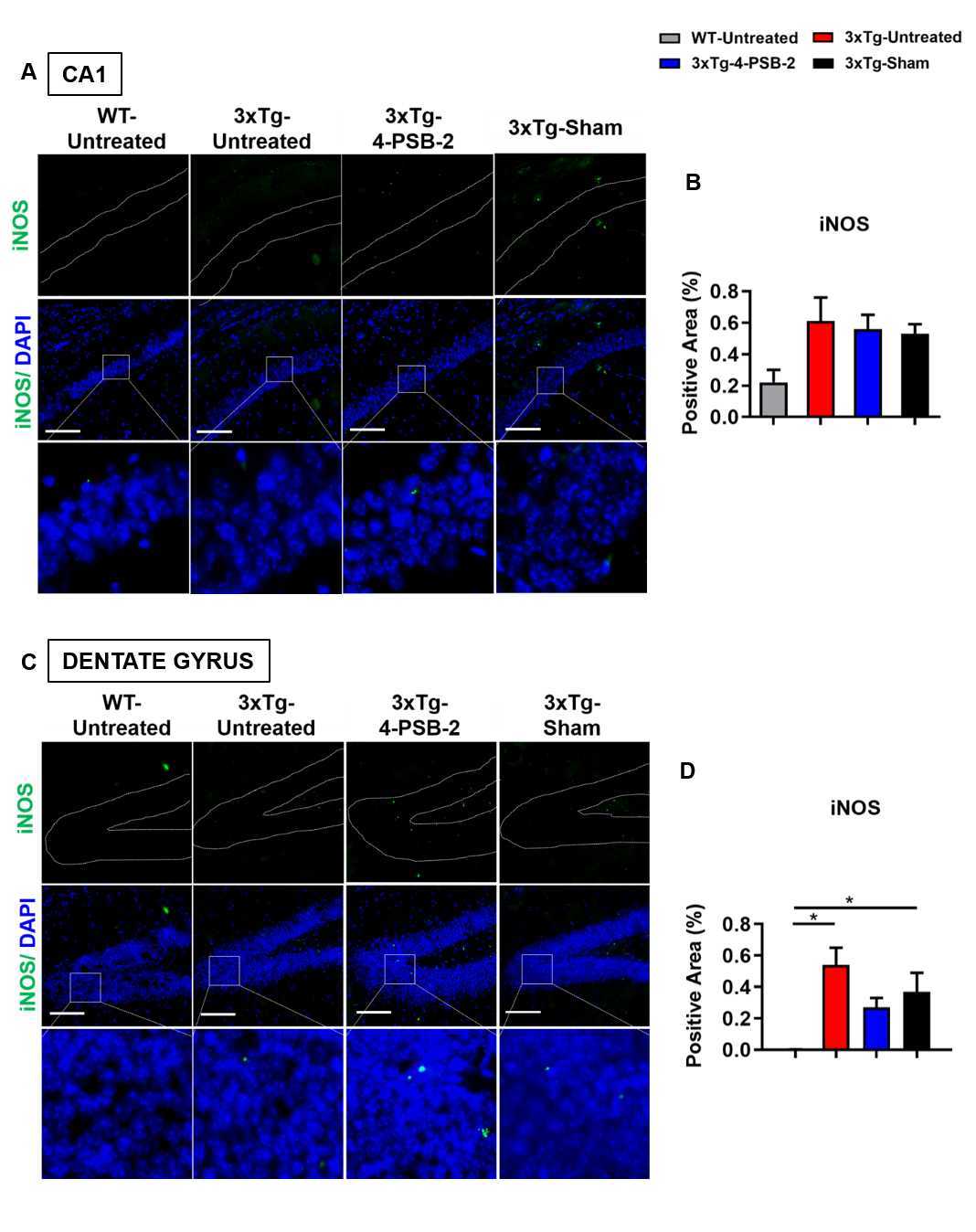


**Supplementary Figure 4.** Expression of iNOS in the hippocampal CA1-region and dentate gyrus of 3xTg-AD mice after TFC. **(A-B)** Immunofluorescence staining and the quantitative results of the hippocampal CA1-region in 3xTg-AD mice showed that the expression levels of iNOS were not significantly different among groups. **(C-D)** The iNOS expression levels in dentate gyrus were significantly increased in the 3xTg-AD-untreated and sham groups. Statistics: one-way ANOVA; **B**, *F*_(3,81)_= 2.503, *p*=0.065; **D**, *F*_(3,48)_= 5.074, *p*<0.01. The results are plotted as the means±SEMs, * indicates *p* ≤0.05, and ** indicates *p*≤ 0.001 between the groups. iNOS (green) and DAPI (blue) = nuclei, Bar= 100 µm.
